# Risk of Malignant Neoplasm in Patients with Primary Hyperparathyroidism: A Systematic Review and Meta-analysis

**DOI:** 10.1007/s00223-024-01219-y

**Published:** 2024-05-21

**Authors:** Nipith Charoenngam, Thanitsara Rittiphairoj, Chalothorn Wannaphut, Watsachon Pangkanon, Sakditat Saowapa

**Affiliations:** 1 Department of Medicine, Harvard Medical School, Mount Auburn Hospital, 330 Mt Auburn St, Cambridge, MA 02138 USA; 2 Department of Medicine, Faculty of Medicine Siriraj Hospital, Mahidol University, Bangkok, Thailand; 3 Department of International Health, Johns Hopkins Bloomberg School of Public Health, Baltimore, MD USA; 4 Division of Health Systems Management, Department of Community Medicine, Faculty of Medicine Ramathibodi Hospital, Mahidol University, Bangkok, Thailand; 5 Department of Medicine, John A. Burns School of Medicine, University of Hawai’i, Honolulu, HI USA; 6 Department of Medicine, Texas Tech University Health Sciences Center, Lubbock, TX USA

**Keywords:** Primary hyperparathyroidism, Malignant neoplasm, Thyroid cancer, Breast cancer, Systematic review, Meta-analysis

## Abstract

This study aimed to evaluate the prevalence and risk of malignant neoplasm in primary hyperparathyroidism (PHPT) patients. Potentially eligible studies were retrieved from PubMed and Embase databases from inception to November 2023 using search strategy consisting of terms for “Primary hyperparathyroidism” and “Malignant neoplasm”. Eligible study must report prevalence of malignant neoplasm among patients with PHPT or compare the risk of malignant neoplasm between patients with PHPT and comparators. Point estimates with standard errors were extracted from each study and combined using the generic inverse variance method.A total of 11,926 articles were identified. After two rounds of systematic review, 50 studies were included. The meta-analysis revealed that pooled prevalence rates of overall cancer was 0.19 (95%CI: 0.13–0.25; *I*^2^ 94%). The two most prevalent types of malignancy among patients with PHPT ware papillary thyroid cancer (pooled prevalence: 0.07; 95%CI: 0.06–0.08; *I*^2^ 85%) and breast cancer (pooled prevalence: 0.05; 95%CI: 0.03–0.07; *I*^2^ 87%). Subgroup analysis of studies focusing on patients undergoing parathyroidectomy reported a fourfold higher prevalence of papillary thyroid cancer than the remaining studies (0.08 versus 0.02). The meta-analysis of cohort studies found a significant association between PHPT and overall cancer with the pooled risk ratio of 1.28 (95%CI: 1.23–1.33; *I*^2^ 66.9%).We found that the pooled prevalence of malignant neoplasm in PHPT was 19%, with papillary thyroid cancer and breast cancer being the most prevalent types. The meta-analysis of cohort studies showed that patient with PHPT carried an approximately 28% increased risk of malignancy.

**Supplementary Information:**

The online version contains supplementary material available at 10.1007/s00223-024-01219-y.

## Introduction

Primary hyperparathyroidism (PHPT) is a disorder of mineral metabolism characterized by increased or unsuppressed levels of parathyroid hormone (PTH) due to excessive secretion from one or more abnormal parathyroid glands [1, 2]. This condition is a relatively common endocrine disorder, with prevalence estimates ranging from 1 to 7 cases per 1,000 adults and an incidence varying from 0.4 to 21.6 cases per 100,000 person-years [3, 4].

There are few established risk factors for PHPT, which include female sex, exposure to ionizing radiation, lithium exposure and genetic predisposition [2]. Over 95% of PHPT cases are sporadic, with less than 5% associated with germline mutations predisposing individuals to the development of parathyroid tumor [5]. Complications of PHPT include hypercalcemia, bone loss and hypercalciuria leading to kidney stones, nephrocalcinosis and kidney failure [1, 2]. Furthermore, evidence from observational studies suggests an association between PHPT and increased risks of medical comorbidities including cardio-metabolic disease and psychiatric disorders [6]. Although the exact causal relationship between PHPT and these conditions are undetermined, it has been thought to be mediated by abnormal calcium homeostasis [7].

Interestingly, studies have suggested a link between PHPT and the risk of developing malignant neoplasm, which is thought to be mediated by different mechanisms including the tumorigenic effects of PTH, abnormal calcium and vitamin D homeostasis, as well as shared genetic and environmental risk factors [8]. From an epidemiological standpoint, multiple studies have revealed varying prevalence rates of various types of malignant neoplasm among patients with PHPT, including thyroid, breast, lung, and colon cancer, among [9–55]. Nevertheless, most of the included studies were performed in a single institution and had small sample sizes, limiting the confidence and generalizability of the findings. Additionally, some studies suggest an increased risk of malignant neoplasm in patients with PHPT compared to those without [45, 56–66]. However, the reported degree of association between PHPT and the risk of malignant neoplasm vary across the studies. Furthermore, it remains unknown whether the risk of malignancy is different in patients with PHPT requiring parathyroidectomy (PTX) compared to mild PHPT, or if the preoperative assessment for PTX leads to increased screening and detection of malignancy, particularly thyroid cancer.

Using a systematic review and meta-analysis technique, the objective of this study is to identify all available data on the prevalence of each type of malignant neoplasm in patients with PHPT, including a subgroup of studies in patients undergoing PTX, and combine them together. Additionally, we aim to determine the pooled effect size of the risk malignant neoplasm in patients with PHPT compared with individuals without PHPT.

## Method

### Search Strategy

Three investigators (C.W., W.P., S.S.) independently conducted searches in the Embase and PubMed databases from inception until November 2023. The search terms were generated from terms related to 'Primary Hyperparathyroidism' and 'Malignant Neoplasm.' The comprehensive search strategy is presented in Supplementary Material 1. There were no restrictions on language. This study adhered to the Preferred Reporting Items for Systematic Reviews and Meta-Analyses (PRISMA) guidelines, as illustrated in Supplementary Material 2.

### Eligibility Criteria

For the meta-analysis of the prevalence of malignant neoplasm in patients with PHPT, eligible studies must include a cohort of patients with PHPT and report the proportion of patients with overall or any type of malignant neoplasm. For the meta-analysis of the risk of malignant neoplasm in patients with PHPT versus comparators without PHPT, eligible studies must include a cohort of patients with PHPT and another cohort of comparators without PHPT. The study must then compare the risk of overall or any type of malignant neoplasm between the two groups. Studies that focused exclusively on patients with parathyroid carcinoma, multiple endocrine neoplasia or genetic forms of PHPT were excluded from both meta-analyses. Additionally, case reports and case series with sample sizes of less than 5 were excluded from the meta-analyses. In cases where multiple studies utilized the same database, only the one with a more comprehensive inclusion of data would be included to avoid duplication of data points.

The retrieved articles were independently evaluated for eligibility by two investigators (N.C and C.W.). Disagreements were resolved through discussion with the methodologist (T.R.). The quality of each included cohort study was assessed by two investigators (N.C., T.R.) using the Newcastle–Ottawa quality assessment scale for cohort study [67].

### Data Extraction

A standardized collection form was employed for data extraction. The collection form included the following information: last name of the first author, year of publication, country of study, number of PHPT patients, the number/proportion of each malignant neoplasm, study institution/data source, whether or not included patients underwent parathyroidectomy, mean age of patients, the percentage of female patients and mean serum PTH and serum calcium. For studies comparing the risk of malignant neoplasm in PHPT patients versus comparators, additional variables were extracted, including number of comparators, diagnosis of PHPT, diagnosis of malignant neoplasm, follow-up criteria, variables adjusted in the multivariate analysis, and effect estimates representing the relative risk of overall and each type of malignant neoplasm.

### Statistical Analysis

All data analyses were conducted using the StataMP15. Proportions with standard errors for count data were extracted from each included study. The pooled effect sizes and 95% confidence interval were computed using DerSimonian and Laird’s generic inverse variance method [68]. A random-effect model was employed instead of fixed-effect model given the diverse background populations and protocols across the included studies. Statistical heterogeneity was assessed using the I^2^ statistics, where I^2^ values of 0–25% indicated insignificant heterogeneity, 26–50% low heterogeneity, 51–75% moderate heterogeneity and > 75% high heterogeneity [69]. In the meta-analysis of prevalence of malignant neoplasm in patients with PHPT, subgroup analyses were performed to explore if studies focusing in patients undergoing PTX yielded different results from the remaining of the studies. Sensitivity analyses were performed by excluding each of the studies with overlapping data to assess the robustness of the results.

## Results

### Search Results

A total of 11,926 articles were initially retrieved from the EMBASE and PubMed databases. After removing 2253 duplicated articles, 11,926 articles remained for title and abstract review. At this stage, 11,789 articles were excluded as they did not meet the eligibility criteria based on article type and study design. This exclusion left 137 potentially eligible articles for full-text review. Subsequently, 79 articles were further excluded due to the lack of the outcome of interest, resulting in 58 articles that fulfilled the eligibility criteria.

Among these 58 eligible articles, 47 reported the prevalence of malignant neoplasm among patients with PHPT [9–55], while 12 compared the risk of malignant neoplasm between patients with PHPT and comparators [45, 56–66]. One study [55] was excluded from the meta-analysis as it reported the prevalence of thyroid cancer in a PHPT patient cohort that was a subset of another study [37]. Seven studies utilized the Swedish Cancer Registry database to report the risk of cancer among PHPT patients in comparison with the general Swedish population [56, 60–65]. Therefore, only one study with the most comprehensive inclusion of data from 1958 to 2008 was included in the meta-analysis [56]. Additionally, two studies [59, 66] utilized the database of residents of Tayside, Scotland; therefore, the one with the more comprehensive inclusion of data from 1997 to 2019 was included [59].

**Fig. 1:**
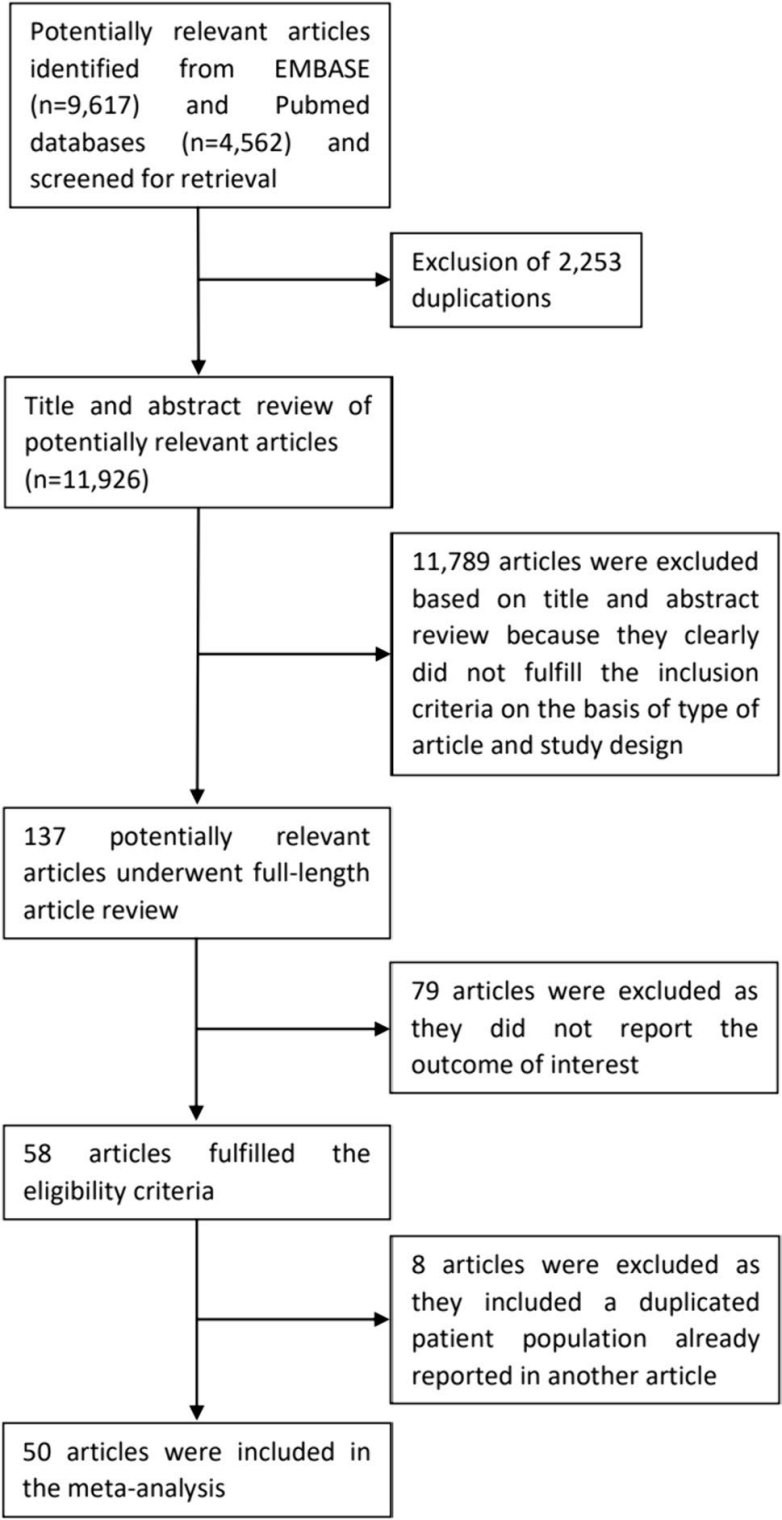
Study identification and literature review process

Finally, a total of 50 articles were included in the meta-analysis. Out of these, 46 were included in the meta-analysis of the prevalence of malignant neoplasm in PHPT [9–54], and 5 were included in the meta-analysis comparing the risk of malignant neoplasm in patients with PHPT versus comparators [45, 56–59]. Figure 1 demonstrates the study identification and literature review process. The characteristics of the eligible studies are shown in Tables 1 and 2.

**Table 1:** Characteristics of studies reporting prevalence of malignant neoplasm in patients with primary hyperparathyroidism

Study	Reference No	Year	Country	Study institution/data source	Recruitment year	PTX patients	Number of patients	Mean age (year)	% Female	Mean PTH (pg/mL)	Mean Ca (mg/dL)
Agbaht et al.	[9]	2010	Turkey	Ankara University Faculty of Medicine	2005–2009	no	145	54.1	82.8	NA	NA
Altinyay et al.	[10]	2012	Saudi Arabia	The National Guard Health Affair King Abdulaziz Medical City	2007–2012	yes	38	NA	NA	NA	NA
Arcieco et al.	[11]	2012	USA	Walter Reed Army Medical Center and Johns Hopkins University Hospital	2005–2008	yes	94	NA	NA	118	11.0
Attie et al.	[12]	1993	USA	Long Island Jewish Medical Center	1953–1992	yes	948	NA	NA	NA	NA
Baykan et al.	[13]	2022	Turkey	Erzurum Regional Training and Research Hospital	2015–2021	yes	255	52	83.9	NA	NA
Bernal et al.	[14]	2014	Spain	Hospital Universitario 12 de Octubre	NA	yes	103	60.2	79.6	NA	NA
Calcaterra et al.	[15]	1980	USA	UCLA School of Medicine	NA	yes	144	NA	NA	NA	NA
Çalişkan et al.	[16]	2023	Turkey	Düzce Atatürk State Hospital	2012–2015	yes	172	54.3	85.5	179	11.1
Castellano et al.	[17]	2018	Italy	Santa Croce and Carle Hospital	1998–2017	no	434	NA	NA	NA	NA
Cetin et al.	[18]	2019	Turkey	Kartal Dr. Lutfi Kirdar Training and Research Hospital	2014–2017	yes	275	55	85.5	NA	NA
Cinamon et al.	[19]	2006	Canada	Jewish General Hospital, McGill University Health Centre and the Maisonneuve–Rosemont Hospital	1992–2004	no	582	60.1	66.0	NA	NA
Farr et al.	[20]	1973	USA	Memorial Sloan–Kettering Cancer Center	1937–1973	yes	100	NA	NA	NA	NA
Fedorak et al.	[21]	1994	USA	University of Chicago, Pritzker School of Medicine	NA	yes	100	NA	NA	NA	NA
Garner et al	[22]	2007	USA	Cleveland clinic	2000–2006	no	1352	NA	NA	NA	NA
Gedik et al.	[23]	2009	Turkey	Dokuz Eylul University Medical Faculty	1980–2007	no	132	50.8	82.6	NA	NA
Gul et al.	[24]	2009	Turkey	Ankara Ataturk Education and Research Hospital	1980–2007	yes	32	NA	NA	NA	NA
Haciyanli et al.	[25]	2022	Turkey	Izmir Kâtip Celebi University Ataturk Training and Research Hospital	2014–2019	yes	284	NA	NA	NA	NA
Heizmann et al.	[26]	2009	Switzerland	Basel University hospital	2003–2006	yes	30	65	56.7	NA	NA
Hickey et al.	[27]	1991	USA	University of Texas M. D. Anderson Cancer Center	1976–1988	no	103	NA	NA	NA	NA
Hu et al.	[28]	2023	China	Wuhan Union Hospital	2016–2021	yes	99	51	78.8	302	10.7
Jeong et al.	[29]	2020	Korea	Seoul St. Mary’s Hospital and Yeouido St. Mary’s Hospital	2009–2019	yes	154	54.7	82.5	207	10.8
Jovanovich et al.	[30]	2017	Serbia	Center for Endocrine Surgery, Clinical Center of Serbia	2008–2015	yes	849	NA	NA	NA	NA
Kambouris et al.	[31]	1987	USA	Henry Ford Hospital	1980–1983	yes	111	57	76.5	NA	NA
Karakose et al.	[32]	2021	Turkey	Necmettin Erbakan University Meram Medical Schoo	2006–2019	no	775	58	80.6	173	11.2
Katluturk et al.	[33]	2014	Turkey	Inonu University Faculty of Medicine	2009–2013	yes	46	52.8	84.8	586	12.1
Kosem et al.	[34]	2004	Turkey	Yüzüncü Yıl University, School of Medicine	1999–2002	yes	51	47.8	86.3	NA	NA
Krause et al.	[35]	1996	Germany	University Clinics of Essen	1979–1993	yes	322	NA	NA	NA	NA
Lehwald et al.	[36]	2013	Germany	University Hospital Moorenstrasse	1986–2012	no	1464	NA	74.1	NA	NA
Li et al.	[37]	2021	China	Qilu Hospital and Yantai Yuhuangding Hospital of Qingdao University	2010–2020	yes	318	53	70.8	285	11.9
Linos et al.	[38]	1982	USA	Mayo Clinic, Rochester	1965–1979	yes	2058	NA	NA	94	11.7
LiVolsi et al.	[39]	1976	USA	Columbia Presbyterian Medical Center	1911–1975	yes	471	NA	NA	NA	NA
Martins et al.	[40]	2019	Portugal	Instituto Portugues de Oncologia de Lisboa Francisco Gentil	2000–2018	yes	188	59	80.3	186	11.5
Masatsugu et al.	[41]	2005	Japan	Noguchi Thyroid Clinic and Hospital Foundation	1998–2001	yes	109	57.4	88.1	139	11.2
Morita et al.	[42]	2008	USA	Johns Hopkins Endocrine Surgery Section	2006–2007	yes	200	NA	NA	NA	NA
Ogawa et al.	[43]	2007	Japan	University of Tokyo	1994–2005	yes	85	57	77.6	206	11.2
Ozkul et al.	[44]	2014	Turkey	Çanakkale Onsekiz Mart University	2006–2012	yes	98	NA	87.8	425	12.3
Palmieri et al.	[45]	2017	Italy	IRCCS Cà Granda–Ospedale Maggiore Policlinico Milan	2014–2016	no	163	NA	NA	124	10.7
Preda et al.	[46]	2019	Romania	“Sf. Spiridon” Emergency Hospital	2010–2017	yes	140	54.6	89.3	358	NA
Regal et al.	[47]	1999	Spain	Endocrine Division Hospital “Xeral–Cíes” of Vigo	1990–1997	yes	54	61	75.9	248	11.6
Rivo Vazquez et al.	[48]	2007	Spain	Complejo Hospitalario Universitario de Vigo	1998–2006	yes	115	NA	NA	NA	NA
Sidhu et al.	[49]	2000	Australia	Liverpool Hospital	1993–1998	yes	65	59	67.7	160	11.6
Simsek et al.	[50]	2017	Turkey	Ankara Numune Training and Research Hospital, Ankara	NA	yes	202	57.8	88.1	NA	NA
Strichartz et al.	[51]	1990	USA	UCLA School of Medicine	NA	yes	52	NA	NA	NA	NA
Vargas–Ortega et al.	[52]	2018	Mexico	Hospital de Especialidades	2013–2015	yes	59	54	78.0	147	11.4
Vita et al.	[53]	2019	Romania	Emergency County Hospital Timisoara	2011–2018	yes	66	54.2	81.8	NA	NA
Xue et al.	[54]	2016	China	Shanghai Tongji Hospital and the First Affiliated Hospital of Nanjing Medical University	2009–2014	yes	155	55	75.5	269	11.6

*Ca* Calcium, *NA* Not available, *PTX* Parathyroidectomy, *PTH* Parathyroid hormone, *Ref No* Reference number

**Table 2:** Main characteristics of the studies comparing risks of malignant neoplasms between patients with primary hyperparathyroidism and comparators

	Fallah et al. [56]	Ghosh et al. [57]	Ogard et al. [58]	Palmieri et al. [45]	Soto-Pedre et al. [59]
Country	Sweden	Scotland	Denmark	Italy	Scotland
Year of publication	2011	2009	2003	2017	2023
Total number of participants	Patients with PHPT: 12,037 Comparators: NA	Patients with PHPT: 3,039 Comparators: NA	Patients with PHPT: 1,578 Comparators: NA	Patients with PHPT: 163 Comparators: 1,443	Patients with PHPT: 6,795 Comparators: 20,385
Recruitment of participants	Cases were patients with parathyroid adenoma identified from the Swedish Cancer Registry during 1958 – 2008 Comparators were general Swedish population	Cases were patients with PHPT identified from the Scottish Cancer Registry Comparators were general Scottish population	Cases were patients with PHPT identified from the National Hospital Patients Register during 1977 – 1993 Comparators were general Danish population	Cases were patients with PHPT attending the “Osteoporosis and Metabolic Disease” outpatients clinic at IRCCS Cà Granda-Ospedale Maggiore Policlinico Milan during May 2014—May 2016 Comparators were patients without PHPT attending the same clinic	Cases were patients with mild PHPT in Tayside, Scotland identified from the Community Health Index at the Health Information Center, University of Dundee during 1997 – 2019 Comparators were patients identified from the same database 1:3 matched for age and sex
Diagnosis of PHPT	Presence of morphologically verified diagnosis of parathyroid adenoma in the Swedish Cancer Registry database	NA	Presence of diagnostic codes for PHPT (ICD-8: 252.00 and 226.89) or those who had undergone parathyroid surgery (national code: 0.84xx)	Presence of diagnosis of PHPT in the medical record	Presence of elevated serum calcium and one or more of the following criteria: increased serum PTH; increased 24-h urinary calcium excretion; histologically proven parathyroid tumor; positive Sestamibi Tc-99 scan results; hospital admission with PHPT diagnosis or record of PTX
Diagnosis of malignant neoplasm	Presence of morphologically verified diagnosis of malignant neoplasm in the Swedish Cancer Registry database	NA	NA	Presence of diagnosis of malignant neoplasm in the medical record	NA
Follow-up criteria	Until death, detection of a primary cancer, emigration or December 2008, whichever came first	NA	Until death or end of December 1993	NA	Until occurrence of clinical outcome of interest or end of study
Mean serum calcium	NA	NA	NA	Cases: 10.7 mg/dL Comparators: 9.5 mg/dL	Cases: 10.8 mg/dL Comparators: 9.2 mg/dL
Mean serum PTH	NA	NA	NA	Cases: 124 pg/mL Comparators: 24 pg/mL	Cases:73 pg/mL Comparators: 60 pg/mL
Variables adjusted in the multivariate analysis	NA	NA	NA	NA	Age, sex, baseline cancer, serum 25-hydroxyvitamin D
Newcastle–Ottawa score	Selection: 4 Comparability: 0 Outcome: 2	Selection: 3 Comparability: 0 Outcome: 1	Selection: 4 Comparability: 0 Outcome: 2	Selection: 3 Comparability: 0 Outcome: 1	Selection: 4 Comparability: 2 Outcome: 3

*ICD*-8 The International Classification of Disease, 8th Revision; *PHPT*: Primary hyperparathyroidism; *PTH* Parathyroid hormone, *PTX* Parathyroidectomy

### Prevalence of Malignant Neoplasm in Patients with Primary Hyperparathyroidism

Among the 46 prevalence studies included in the meta-analysis, 37 studies were conducted on PHPT patients who underwent PTX. The mean age ranged from 48 to 65 years and the percentage of female participants ranged from 57–89%. The mean ± SD of serum PTH levels across studies was 234 ± 124 pg/mL (from 20 studies), while the mean ± SD of serum calcium levels was 11.4 ± 0.5 mg/dL (from 19 studies). The results of meta-analysis of prevalence of malignant neoplasm in patients with PHPT are presented in Table 3. Based on the results of 9 studies involving a total of 2,377 PHPT patients, the pooled prevalence of overall cancer was 19% (95%CI: 13–25%), with *I*^2^ of 94% indicating high statistical heterogeneity. The most prevalent and frequently reported type of malignant neoplasm among patients with PHPT was thyroid cancer (any type) (46 studies; 13,792 PHPT patients; pooled prevalence: 7%; 95%CI: 6–9%; *I*^2^ = 85%), followed by papillary thyroid cancer (32 studies; 9495 patients; pooled prevalence: 7%; 95%CI: 6–8%; *I*^2^ = 85%) and breast cancer (8 studies; 3374 patients; 5%; 95%CI: 3–7%; *I*^2^ = 87%). The less frequently reported cancers included lung cancer, gynecological cancer, kidney cancer, colon cancer, prostate cancer, urinary tract cancer, skin basal cell carcinoma, hematologic malignancy and gastric cancer, with pooled prevalence values ranging between 0–2% (Table 3). List of studies included in the meta-analysis is presented in Supplementary material 3.

**Table 3:** Meta-analysis of prevalence of malignant neoplasms among patients with primary hyperparathyroidism

Type of malignant neoplasm	All studies				Subgroup of studies of PHPT patients undergoing PTX				Subgroup of studies of PHPT patients regardless of PTX status				p-subgroup difference
	No. of studies	No. of PHPT patients	Pooled prevalence (95%CI)	*I*^2^ (%)	No. of studies	No. of PHPT patients	Pooled prevalence (95%CI)	*I*^2^ (%)	No. of studies	No. of PHPT patients	Pooled prevalence (95%CI)	I^2^ (%)	
Overall cancer	9	2377	19% (13–25%)	94	5	754	18% (8–28%)	95	4	1623	21% (13–29%)	94	0.68
Thyroid cancer (any type)	46	13,792	7% (6–9%)	85	37	8642	9% (7–10%)	86	9	5150	5% (3–6%)	85	< 0.001
Papillary thyroid cancer	32	9495	7% (6–8%)	85	28	7,320	8% (6–10%)	85	4	2175	2% (2–3%)	9	< 0.001
Breast cancer	8	3374	5% (3–7%)	87	3	399	6% (1–11%)	32	5	2975	4% (2–7%)	91	0.6
Lung cancer	5	1059	1% (0–2%)	0	2	211	1% (0–2%)	–	3	848	1% (0–2%)	–	0.76
Gynecological cancer	5	1723	0% (0–1%)	3	1	100	1% (0–5%)	–	4	1623	0% (0–1%)	18	0.53
Kidney cancer	5	1731	0% (0–1%)	0	2	211	0% (0–2%)	–	3	1520	0% (0–1%)	–	0.47
Colon cancer	4	956	1% (0–3%)	60	2	211	4% (2–7%)	–	2	745	1% (0–1%)	–	0.01
Prostate cancer	4	1708	1% (0–1%)	–	1	188	2% (0–5%)	–	3	1520	1% (0–1%)	–	0.28
Hematologic malignancy	4	476	2% (1–3%)	9	3	373	2% (0–3%)	–	1	103	3% (1–8%)	–	0.53
Urinary tract cancer	3	796	1% (0–2%)	–	1	111	1% (0–5%)	–	2	685	1% (0–4%)	–	0.91
Skin basal cell carcinoma	3	1066	1% (0–1%)	–	1	188	2% (1–5%)	–	2	878	1% (0–1%)	–	0.22
Gastric cancer	3	986	2% (0–5%)	–	2	211	2% (0–3%)	–	1	775	1% (0–2%)	–	0.49

95%CI: 95% Confidence interval;

*No* Number, *PHPT* Primary hyperparathyroidism, *PTX* Parathyroidectomy

### Subgroup Analysis of Patients Undergoing Parathyroidectomy

The subgroup analysis of studies focusing on PHPT patients undergoing PTX revealed pooled prevalence rates of thyroid cancer (any type), and papillary thyroid cancer of 9% (95%CI: 7–10%) and 8% (95%CI: 6–10%), respectively. These values were statistically significantly higher than the pooled prevalence rates based on studies that included PHPT patients regardless of PTX status (thyroid cancer, any type: 5%; 95%CI: 3–6%; papillary thyroid cancer: 2%; 95%CI: 2–3%, *p*-value for subgroup difference both < 0.001). In addition, the pooled prevalence of colon cancer reported in studies of patients undergoing PTX was statistically significantly higher that of the remaining studies (4%; 95%CI: 2–7% versus 1%; 95%CI: 0–1%, *p*-value for subgroup difference = 0.01), although the number of included studies was limited to 2 for both subgroups.

### Risk of Malignant Neoplasm in Patients with Primary Hyperparathyroidism Versus Comparators

**Fig. 2:**
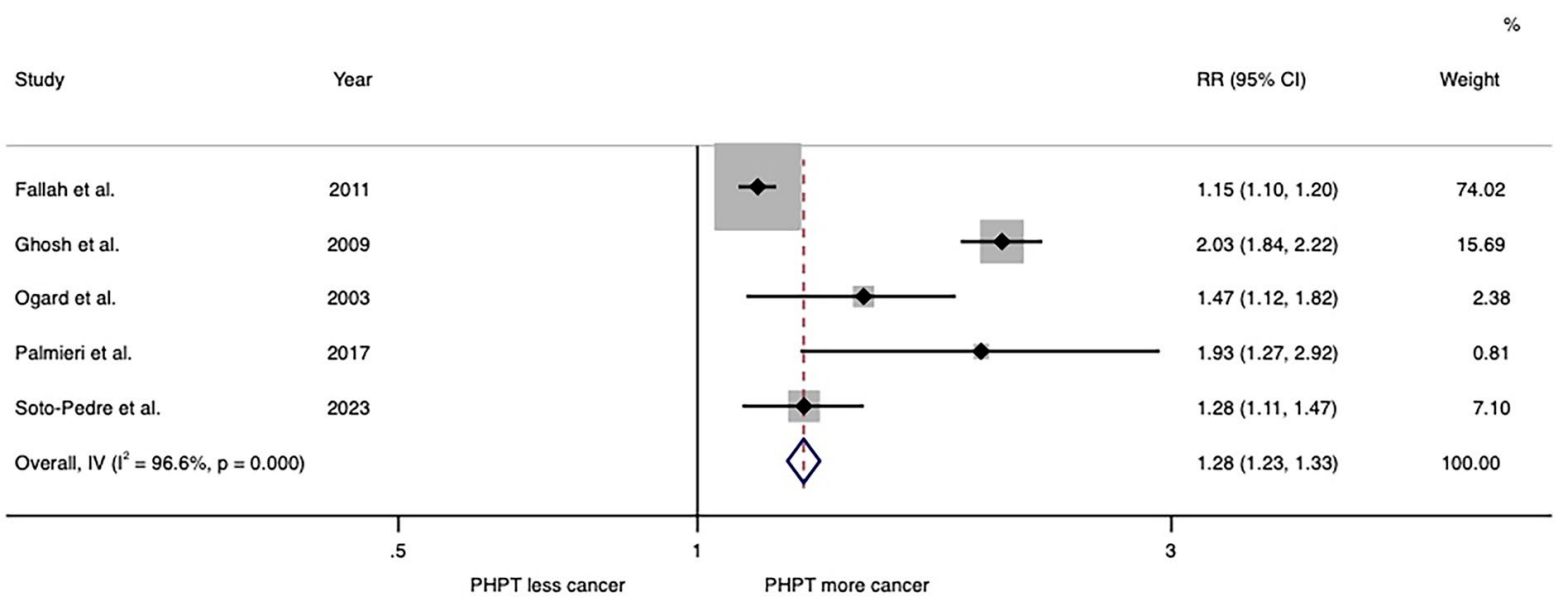
Forest plot of the meta-analysis of risk of overall cancer in patients with primary hyperparathyroidism versus comparators

The meta-analysis of 5 studies found a significant association between PHPT and risk of all cancers with the pooled risk ratio of 1.28 (95%CI: 1.23–1.33), as shown in Fig. 2. This meta-analysis had moderate statistical heterogeneity with *I*^2^ of 66.9%. The two studies by Ghosh et al. [57] and Soto-Pedre et al. [59] utilized databases of the Scottish population. This might have resulted in duplication of included participants. Therefore, a sensitivity analysis was performed by removing one of these two studies. The pooled risk ratio became slightly lower after excluding Ghosh et al.’s study [57] (1.17, 95%CI: 1.13 – 1.22; *I*^2^ of 73.0%) and remained the same after excluding Soto-Pedre et al.’s study (1.28, 95%CI: 1.23 – 1.33; *I*^2^ of 97.5%) [59].

Note that only two studies [45, 56] compared the risks of specific types of malignant neoplasm in patients with PHPT versus comparators; therefore, meta-analysis was not performed. Fallah et al.’s study [56] revealed that benign parathyroid tumor was statistically significantly associated with increased risks of primary cancers of the small intestine (standardized incidence ratio, SIR 2.28, 95%CI: 1.33–3.64), breast (SIR 1.20, 95%CI: 1.08–1.34), kidney (SIR 1.84, 95%CI: 1.45–1.20), melanoma (SIR 1.44, 95%CI: 1.12–1.81), nervous system (1.60, 95%CI: 1.23–2.05), thyroid (SIR 3.15, 95%CI: 2.17–4.42) and polycythemia vera (SIR 2.03, 95%CI: 1.05–3.55). Palmieri et al.’s study [45] revealed that PHPT was statistically significantly associated with cancers of the breast (odds ratio, OR 1.93, 95%CI: 1.13–3.31), kidney (OR 9.05, 95%CI: 2.24–36.54) and skin (OR 6.74, 95%CI: 1.50–30.41).

## Discussion

This is the first systematic review and meta-analysis that compiles all available evidence on malignant neoplasm in patients with PHPT. Our pooled analysis showed that the prevalence of any type of malignant neoplasm among PHPT patients was approximately 20%. Papillary thyroid cancer and breast cancer were the two most common and frequently reported types of malignant neoplasm with the pooled prevalence rates of 7% and 5%. These prevalence rates are considered disproportionately higher than those of the global population, given the global age-standardized incidence rates of thyroid cancer of 2.05 cases per 100,000 population in 2019 and breast cancer of 100.9 cases per 100,000 population in 2020 based on data from the Global Burden of Disease (GBD) database [70, 71].

Other less prevalent types of malignant neoplasm included lung cancer, gynecological cancer, kidney cancer, colon cancer, prostate cancer, urinary tract cancer, skin basal cell carcinoma, hematologic malignancy and gastric cancer, with pooled prevalence rates of 0–2%. In addition, the meta-analysis of 5 cohort studies utilizing population databases from European countries revealed that patients with PHPT carried approximately 28% increased risk of malignancy when compared with comparators.

These observations may suggest the link between PHPT and tumor development outside of known tumors associated with multiple endocrine neoplasia. There are a number of possible explanations to these findings. The first perceivable explanation is selection bias as the rate of screening and detection of cancer may be increased as a result of clinical care in patients with PHPT. This is particularly true in the case of thyroid cancer. As thyroid ultrasound or neck CT scan are parts of routine evaluation in patients with PHPT undergoing PTX [72], those with concurrent asymptomatic thyroid cancer may have been screened and diagnosed through this process. This hypothesis can be supported by the result of our subgroup meta-analysis showing a fourfold higher pooled prevalence rate of papillary thyroid cancer in studies focusing on patients undergoing PTX compared with studies that included PHPT patients regardless of PTX status (8% versus 2%). It is also possible that the increased pooled prevalence rate of breast cancer was secondary to increased cancer screening in the studied population.

In addition to selection bias, there are a number of biological explanations connecting PHPT to the development of malignant neoplasm. First, experimental studies have demonstrated that increased PTH-1 receptor signaling can lead to increased cell proliferation and survival in various cancer cell types, including breast cancer, prostate cancer and renal cell carcinoma [73]. Second, it is conceivable that PHPT and non-parathyroid tumors may share certain risk factors. For instance, neck radiation, a well-known risk factor for thyroid cancer, has been shown in multiple studies to contribute to the development of parathyroid tumor [74]. It is also important to note that approximately 5–10% of clinically diagnosed multiple endocrine neoplasia type 1 (MEN1) patients have negative genetic test results [75] and that MEN1 has been proposed to predispose individuals to the development of breast cancer [76]. This could potentially explain the observed increased risk of breast cancer in patients with PHPT, even after excluding studies focusing on patients with MEN1. However, it remains to be elucidated whether there is a shared genetic predisposition between PHPT and malignant neoplasm outside of known genetic forms of PHPT. Finally, vitamin D deficiency, characterized by low levels of serum 25-hydroxyvitamin D [25(OH)D] concentration, is known to be common in PHPT due to increased inactivation of 25(OH)D driven by increased 1,25-dihydroxyvitamin D [1,25(OH)_2_D] [77, 78]. Numerous observational studies have revealed association between vitamin D deficiency and an increased risk of cancer [80]. This association is believed to be explained by the antiproliferative and prodifferentiative effects of vitamin D receptor activation in various cancer cells [79]. However, evidence from large-scale clinical trials does not support a causal association between vitamin D supplementation regardless of baseline vitamin D status in the general population and a reduction in cancer risk [81, 82].

This study has certain limitations that warrant acknowledgment. First, while several studies on the prevalence of malignant neoplasms were identified, our study's pooled prevalence results only reflect the prevalence reported in the included studies, with participants having a mean age in the 50s-60s and being predominantly women. As a result, the lifetime risk of malignant neoplasms cannot be extrapolated from our findings. Second, the number of included cohort studies was limited, and all studies were conducted in European countries. This limitation restricts the generalizability of our results to the global population. Additionally, data on the relative risk of specific types of malignant neoplasms were scarce, with information available only from 2 studies [45, 56]. Furthermore, there was no data from cohort studies assessing the impact of PTX on malignancy risk. Moreover, the meta-analysis showed moderate to high statistical heterogeneity. Differences in participant characteristics and study design likely served as the primary contributors to the observed heterogeneity. Finally, due to the limited number of included cohort studies, it was not possible to evaluate publication bias using funnel plots.

## Conclusion

The current systematic review and meta-analysis compiled all existing evidence regarding malignant neoplasms in patients with PHPT. The pooled analysis showed that the prevalence of any form of malignant neoplasm in PHPT patients was around 20%. Notably, papillary thyroid cancer and breast cancer emerged as the most prevalent types, with pooled prevalence rates of 7% and 5%, respectively. These rates are considered disproportionately higher than the rates reported in the general population, which may suggest a new clinical entity that requires further investigations. Notably, the high prevalence of thyroid cancer is likely attributed to increased screening and diagnosis rates a part of the preoperative evaluation for PTX, since the prevalence was approximately 4 times higher among studies focusing on PHPT patients undergoing PTX. Furthermore, a meta-analysis of 5 cohort studies demonstrated an approximately 28% increased risk of malignancy in patients with PHPT compared with comparators.

### Supplementary Information

Below is the link to the electronic supplementary material.

Supplementary material 1 (DOCX 20.9 kb)

Supplementary material 2 (DOCX 72.0 kb)

Supplementary material 3 (DOCX 58.8 kb)
