# Supplementary material for: Risk of Malignant Neoplasm in Patients with Primary Hyperparathyroidism: A Systematic Review and Meta-analysis
Source: Calcif Tissue Int. 2024 May 21;115(1):1–13. doi: 10.1007/s00223-024-01219-y (PMC11153283; doi:10.1007/s00223-024-01219-y)
Supplement: Supplementary file 1 — Supplementary material 1 (DOCX 20.9 kb) [file 223_2024_1219_MOESM1_ESM.docx]

**Supplementary material 1** – Search strategy

**EMBASE Database**

1. 'primary hyperparathyroidism'/exp OR 'primary hyperparathyroidism'
2. 'parathyroid adenoma'/exp OR 'parathyroid adenoma'
3. 'hyperparathyroidism'
4. 'malignancy'/exp OR 'malignancy'
5. 'malignant neoplasm'/exp OR 'malignant neoplasm'
6. 'cancer'/exp OR 'cancer'
7. #1 OR #2 OR #3
8. #4 OR #5 OR #6
9. #7 AND #8

**Pubmed Database**

((((((cancer[Title/Abstract]) OR (malignancy[Title/Abstract])) OR (carcinoma[Title/Abstract])) OR (tumor[Title/Abstract])) OR (tumour[Title/Abstract])) OR (neoplasm[Title/Abstract])) AND ((hyperparathyroidism[Title/Abstract]) OR (primary hyperparathyroidism[Title/Abstract]))
