# Supplementary material for: Risk of Malignant Neoplasm in Patients with Primary Hyperparathyroidism: A Systematic Review and Meta-analysis
Source: Calcif Tissue Int. 2024 May 21;115(1):1–13. doi: 10.1007/s00223-024-01219-y (PMC11153283; doi:10.1007/s00223-024-01219-y)
Supplement: Supplementary file 3 — Supplementary material 3 (DOCX 58.8 kb) [file 223_2024_1219_MOESM3_ESM.docx]

**Supplementary material 3** – List of studies included in the mata-analysis of prevalence of malignant neoplasm in patients with primary hyperparathyroidism

| **Study** | **Ref No.** | **Year** | **PTX patients** | **Any cancer** | **Any thyroid cancer** | **Papillary thyroid cancer** | **Breast cancer** | **Lung cancer** | **Gynecological cancer** | **Renal cancer** | **Colon cancer** | **Prostate cancer** | **Hematologic malignancy** | **Urinary tract cancer** | **Basal cell carcinoma** | **Gastric cancer** |
| --- | --- | --- | --- | --- | --- | --- | --- | --- | --- | --- | --- | --- | --- | --- | --- | --- |
| Agbaht et al. | 9 | 2010 | no |  | X | X |  |  |  |  |  |  |  |  |  |  |
| Altinyay et al. | 10 | 2012 | yes |  | X |  |  |  |  |  |  |  |  |  |  |  |
| Arcieco et al. | 11 | 2012 | yes |  | X | X |  |  |  |  |  |  |  |  |  |  |
| Attie et al. | 12 | 1993 | yes |  | X | X |  |  |  |  |  |  |  |  |  |  |
| Baykan et al. | 13 | 2022 | yes |  | X |  |  |  |  |  |  |  |  |  |  |  |
| Bernal et al. | 14 | 2014 | yes |  | X | X |  |  |  |  |  |  |  |  |  |  |
| Calcaterra et al. | 15 | 1980 | yes |  | X |  |  |  |  |  |  |  |  |  |  |  |
| Çalişkan et al. | 16 | 2023 | yes |  | X | X |  |  |  |  |  |  |  |  |  |  |
| Castellano et al. | 17 | 2018 | no |  | X | X |  |  |  |  |  |  |  |  |  |  |
| Cetin et al. | 18 | 2019 | yes |  | X | X |  |  |  |  |  |  |  |  |  |  |
| Cinamon et al. | 19 | 2006 | no | X | X |  | X | X | X | X | X | X |  | X |  |  |
| Farr et al. | 20 | 1973 | yes | X | X |  | X | X | X | X | X |  | X |  |  | X |
| Fedorak et al. | 21 | 1994 | yes |  | X |  |  |  |  |  |  |  |  |  |  |  |
| Garner et al. | 22 | 2007 | no |  | X |  | X |  |  |  |  |  |  |  |  |  |
| Gedik et al. | 23 | 2009 | no |  | X | X |  |  |  |  |  |  |  |  |  |  |
| Gul et al. | 24 | 2009 | yes |  | X | X |  |  |  |  |  |  |  |  |  |  |
| Haciyanli et al. | 25 | 2022 | yes |  | X | X |  |  |  |  |  |  |  |  |  |  |
| Heizmann et al. | 26 | 2009 | yes |  | X | X |  |  |  |  |  |  |  |  |  |  |
| Hickey et al. | 27 | 1991 | no | X | X |  | X | X | X |  |  |  | X | X | X |  |
| Hu et al. | 28 | 2023 | yes |  | X | X |  |  |  |  |  |  |  |  |  |  |
| Jeong et al. | 29 | 2020 | yes |  | X | X |  |  |  |  |  |  |  |  |  |  |
| Jovanovich et al. | 30 | 2017 | yes |  | X | X |  |  |  |  |  |  |  |  |  |  |
| Kambouris et al. | 31 | 1987 | yes | X | X | X | X | X |  | X | X |  |  | X |  | X |
| Karakose et al. | 32 | 2021 | no | X | X |  | X |  | X | X |  | X |  |  | X | X |
| Katluturk et al. | 33 | 2014 | yes |  | X | X |  |  |  |  |  |  |  |  |  |  |
| Kosem et al. | 34 | 2004 | yes |  | X | X |  |  |  |  |  |  |  |  |  |  |
| Krause et al. | 35 | 1996 | yes |  | X |  |  |  |  |  |  |  |  |  |  |  |
| Lehwald et al. | 36 | 2013 | no |  | X | X |  |  |  |  |  |  |  |  |  |  |
| Li et al. | 37 | 2021 | yes |  | X | X |  |  |  |  |  |  |  |  |  |  |
| Linos et al. | 38 | 1982 | yes |  | X | X |  |  |  |  |  |  |  |  |  |  |
| LiVolsi et al. | 39 | 1976 | yes |  | X | X |  |  |  |  |  |  |  |  |  |  |
| Martins et al. | 40 | 2019 | yes | X | X | X | X |  |  |  |  | X | X |  | X |  |
| Masatsugu et al. | 41 | 2005 | yes |  | X |  |  |  |  |  |  |  |  |  |  |  |
| Morita et al. | 42 | 2008 | yes | X | X | X |  |  |  |  |  |  |  |  |  |  |
| Ogawa et al. | 43 | 2007 | yes |  | X | X |  |  |  |  |  |  | X |  |  |  |
| Ozkul et al. | 44 | 2014 | yes |  | X | X |  |  |  |  |  |  |  |  |  |  |
| Palmieri et al. | 45 | 2017 | no | X | X |  | X | X | X | X | X | X |  |  |  |  |
| Preda et al. | 46 | 2019 | yes |  | X | X |  |  |  |  |  |  |  |  |  |  |
| Regal et al. | 47 | 1999 | yes |  | X | X |  |  |  |  |  |  |  |  |  |  |
| Rivo Vazquez et al. | 48 | 2007 | yes |  | X | X |  |  |  |  |  |  |  |  |  |  |
| Sidhu et al. | 49 | 2000 | yes |  | X | X |  |  |  |  |  |  |  |  |  |  |
| Simsek et al. | 50 | 2017 | yes |  | X |  |  |  |  |  |  |  |  |  |  |  |
| Strichartz et al. | 51 | 1990 | yes |  | X |  |  |  |  |  |  |  |  |  |  |  |
| Vargas–Ortega et al. | 52 | 2018 | yes |  | X | X |  |  |  |  |  |  |  |  |  |  |
| Vita et al. | 53 | 2019 | yes |  | X | X |  |  |  |  |  |  |  |  |  |  |
| Xue et al. | 54 | 2016 | yes | X | X | X |  |  |  |  |  |  |  |  |  |  |
